# Waterlogging-Stress-Responsive LncRNAs, Their Regulatory Relationships with miRNAs and Target Genes in Cucumber (*Cucumis sativus* L.)

**DOI:** 10.3390/ijms22158197

**Published:** 2021-07-30

**Authors:** Kinga Kęska, Michał Wojciech Szcześniak, Adela Adamus, Małgorzata Czernicka

**Affiliations:** 1 Department of Plant Biology and Biotechnology, Faculty of Biotechnology and Horticulture, University of Agriculture in Krakow, Al. 29 Listopada 54, 31-425 Krakow, Poland; a.adamus@urk.edu.pl; 2 Institute of Human Biology and Evolution, Faculty of Biology, Adam Mickiewicz University, Uniwersytetu Poznańskiego 6, 61-614 Poznań, Poland; szczesniak.pl@gmail.com

**Keywords:** cucumber, hypoxia, lncRNA, long-term waterlogging, miRNA, priming

## Abstract

Low oxygen level is a phenomenon often occurring during the cucumber cultivation period. Genes involved in adaptations to stress can be regulated by non-coding RNA. The aim was the identification of long non-coding RNAs (lncRNAs) involved in the response to long-term waterlogging stress in two cucumber haploid lines, i.e., DH2 (waterlogging tolerant—WL-T) and DH4 (waterlogging sensitive—WL-S). Plants, at the juvenile stage, were waterlogged for 7 days (non-primed, 1xH), and after a 14-day recovery period, plants were stressed again for another 7 days (primed, 2xH). Roots were collected for high-throughput RNA sequencing. Implementation of the bioinformatic pipeline made it possible to determine specific lncRNAs for non-primed and primed plants of both accessions, highlighting differential responses to hypoxia stress. In total, 3738 lncRNA molecules were identified. The highest number (1476) of unique lncRNAs was determined for non-primed WL-S plants. Seventy-one lncRNAs were depicted as potentially being involved in acquiring tolerance to hypoxia in cucumber. Understanding the mechanism of gene regulation under long-term waterlogging by lncRNAs and their interactions with miRNAs provides sufficient information in terms of adaptation to the oxygen deprivation in cucumber. To the best of our knowledge, this is the first report concerning the role of lncRNAs in the regulation of long-term waterlogging tolerance by priming application in cucumber.

## 1. Introduction

Plants are constantly exposed to unfavourable environmental factors; therefore, a condition for their survival is the rapid activation of defence mechanisms and the ability to adapt to stressful conditions. Lowering the oxygen content below optimal conditions, referred to hypoxia, is a phenomenon that often occurs in the natural environment of plants [1,2]. Limited availability of oxygen in the root zone negatively affects the metabolism of the whole plant, impairing the growth and development [3]. Plants, however, have evolved adaptive mechanisms, causing changes at the molecular, biochemical and physiological levels, consequently leading to morphological changes that allow the transport of oxygen to the insufficiently oxygenated zones [4].

One of the ways to increase stress tolerance in plants is a priming mechanism, i.e., exposure of plants to stress conditions, leading to changes at the physiological and molecular levels, allowing plants to develop a more effective response when faced with another stress induction [5,6]. In our previous work, by priming application, we depicted six genes, i.e., GDSL esterase/lipase, aspartate aminotransferase (*AspAt*), glutamate decarboxylase (*GAD*), sucrose synthase (*SuSy*), triosephosphate isomerase, cytosolic (*TPI*) and expansin-like A1 (*EXP1*) involved in acquiring tolerance to long-term waterlogging in cucumber [7]. Regulation of those genes can be mediated by non-coding RNAs (ncRNAs) [8,9], which include, among others, micro RNAs (miRNAs), small interfering RNAs (siRNAs) and long non-coding RNAs (lncRNAs) [10].

miRNAs are the best known and most abundant short regulatory RNA molecules in plant and animal cells, with a typical length of 20 to 24 nucleotides. The biogenesis of plant miRNAs takes place almost entirely in the cell nucleus. The miRNA genes are first transcribed, most often by RNA polymerase II, generating primary miRNA transcripts (pri-miRNAs), which are then subjected to catalytic cleavage to produce the so-called pre-miRNA molecules, typically 50 to 100 nucleotides in length [11], forming characteristic secondary structures, dubbed hairpins or stem-loops. In the next stage, the mature miRNA is excised from the pre-miRNA and incorporated into the RISC (RNA-Induced Silencing Complex) silencing complex, which participates in the processes of gene expression regulation [12]. miRNAs control gene expression at the post-transcriptional level by inhibiting mRNA translation or cutting the transcript by the AGO1 protein [13] using extensive complementarity to the target sequence [14,15].

In plants, miRNAs regulate proper tissue differentiation, organ and the vascular system development [16]. The increased expression of miRNA data was also demonstrated in plants subjected to various stress factors, such as drought [17], salinity [18], heat [19], nutrient deficiency [20], or heavy metals [21], which indicate that miRNAs are also involved in the adaptation mechanisms to stressful conditions [22]. When it comes to hypoxia, studies concerning identification of miRNAs were conducted in *Arabidopsis thaliana* [23], *Zea mays* [24], *Populus tomentosa* [25], *Medicago sativa* [26], *Solanum habrochaites* [27] and *Cucumis sativus* in the context of adventitious roots formation [28].

LncRNAs represent another class of regulatory transcripts that participate in response to stresses in plants. They are defined as RNA molecules over 200 bp in length that are not translated into functional proteins. They are mainly located in the cell nucleus, in chromatin fractions, but also with a lower frequency in the cytosol [29]. LncRNAs are tissue-specific molecules with low expression levels and a low level of conservation between species [30].

LncRNAs have been classified according to their location in the genome and the function they play in cells. Taking into account their relative orientation towards proximal protein-coding genes, they can be defined as sense, antisense, bidirectional, intronic, and intergenic lncRNAs [31]. LncRNAs can act as enhancers or repressors of gene expression in either cis or trans regulation in a way [32]. They regulate gene activity on transcriptional, post-transcriptional or translational levels and by interactions with DNA, RNA and proteins [33,34].

LncRNAs are the least understood group of transcripts in the genomes of living organisms. Identification and determination of the functions of individual lncRNAs have so far been widely described, mainly in humans, but also in other animals [35,36,37]. In plants, they were only identified in a few species [38], which indicates the need to extend research in this area. The first work describing the function of lncRNA molecules in plants was published in 2004. The function of lncRNA, known as Enod40 in alfalfa, which is involved in the relocation of the nuclear RNA binding protein to the cytoplasm, was then determined [39]. Subsequent studies have shown that in plants, lncRNAs play a key role in the flowering and reproduction process [40], root organogenesis and seedling photomorphogenesis [41,42]. The lncRNAs expressed in response to biotic and abiotic stresses were also characterized [43,44,45,46].

Cucumber (*Cucumis sativus* L.) is an annual plant from the *Cucurbitaceae* family, characterized by a shallow root system. Throughout the vegetation, cucumber is exposed to several unfavourable environmental factors that lead to limited availability of oxygen [47,48]. One of them is excess water in the soil, which negatively affects the productivity of crops [49,50]. 

Many studies have demonstrated that the process of post-transcriptional regulation of gene expression with the participation of non-coding RNA molecules is crucial in response to environmental changes in plants [51,52], because it enables survival and activates adaptive mechanisms to stress factors [53]. There is no information in the literature on the role of non-coding RNA molecules in the post-transcriptional regulation of genes induced in conditions of oxygen deficiency in the cucumber root zone; hence, it is important to broaden the knowledge in this field. Now, thanks to the rapid advances in deep transcriptome sequencing (RNA-Seq) technology and related bioinformatics methods, in silico tools are available to identify novel non-coding RNA molecules.

The aim of the study was to identify lncRNAs and miRNAs that participate in response to long-term waterlogging stress in cucumber accessions with confirmed diverse tolerance [7,54]. Additionally, an attempt to identify lncRNAs involved in acquiring tolerance to oxygen deprivation in cucumber through priming application has been made. Our goal was also to examine the expression levels of selected ncRNAs at an earlier of response to hypoxia stress (2 days). Interaction between differentially expressed lncRNAs and miRNAs was examined to determine potential regulatory pathways.

## 2. Results

### 2.1. LncRNAs Identified in Cucumber under Long-Term Waterlogging

**Figure 1:**
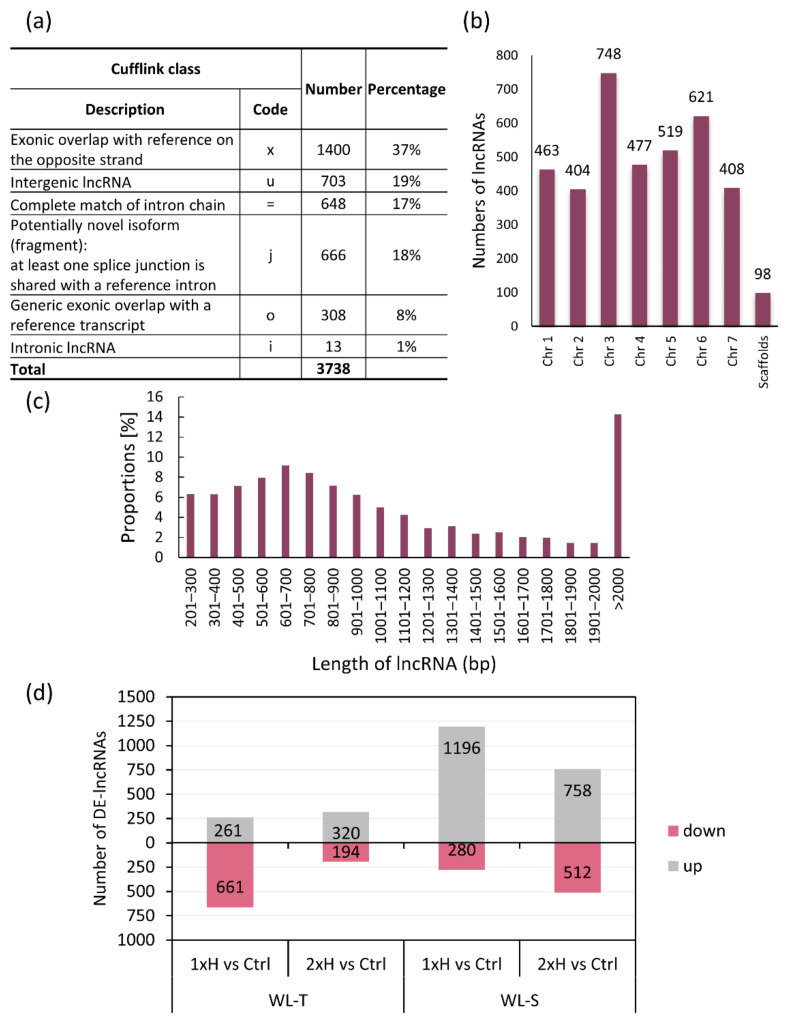
Characterization of lncRNAs in two cucumber accessions (WL-T and WL-S) under long-term waterlogging. (**a**) Classification of lncRNAs on the basis of their genomic locations with respect to adjacent protein coding genes. (**b**) Distribution of lncRNAs across chromosomes. (**c**) Transcript size distributions for all lncRNAs. (**d**) Number of DE-lncRNA (Differentially Expressed lncRNA) identified in each performed comparison with control conditions in both cucumber accessions. WL-T—waterlogging tolerant accession, WL-S—waterlogging sensitive accession, Ctrl—unstressed plants, 1xH—non-primed, once waterlogged plants, 2xH—primed, twice waterlogged plants.

The total number of identified lncRNAs was 3738, which accounted for 10% of all identified transcripts (35712) in de novo assembled transcriptomes for unstressed (Ctrl), non-primed, once waterlogged plants (1xH) and primed, twice waterlogged plants (2xH) [7] (Supplementary Materials Data S1). The largest percentage of lncRNAs (37%) was classified as exonic overlap with reference on the opposite strand (‘x’) and 19% were classified as unknown intergenic transcripts (‘u’) (Figure 1a). Identified lncRNAs were distributed across all cucumber chromosomes, and the highest number of lncRNAs was located on chromosome 3, and later on chromosome 6 (Figure 1b). Approximately 15% of identified lncRNAs were longer than 2000 bp (Figure 1c). 

Analysis of differential expression showed the highest number of DE-lncRNAs (Differentially Expressed lncRNAs) in comparison to control conditions in waterlogging sensitive (WL-S) accession (DH4) after induction of 7 days of waterlogging (1xH), i.e., 1476, of which 81% were up-regulated. In primed plants of WL-S (2xH), the total number of DE-lnRNAs was 1270, while the smallest number of regulated genes were detected in the waterlogging-tolerant (WL-T) accession (DH2) under the second waterlogging treatment (2xH), i.e., 514 (62% of them upregulated) (Figure 1d, Supplementary Materials Data S2–S5). 

**Figure 2:**
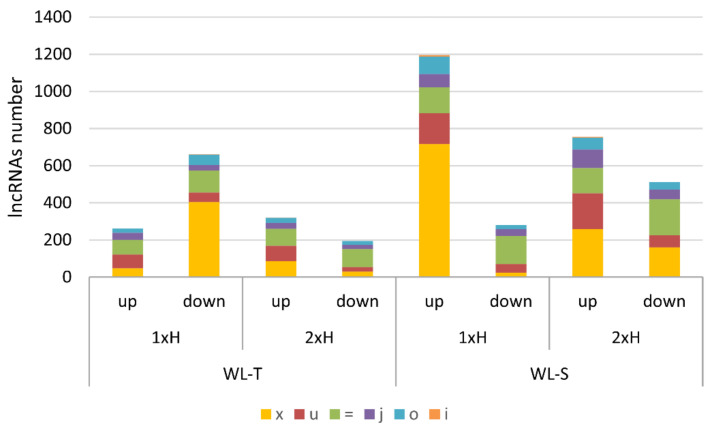
Distribution of lncRNAs due to genome localization among differentially expressed lncRNAs in two cucumber accessions (WL-T and WL-S) under waterlogging (1xH and 2xH) in comparisons to control conditions. ‘x’—exonic overlap with reference on the opposite strand, ‘u’—intergenic lncRNA, ‘=’—complete match of intron chain, ‘j’—potentially novel isoform (fragment): at least one splice junction is shared with a reference intron, ‘o’—generic exonic overlap with a reference transcript, ‘i’—intronic lncRNA.

We also determined the number of DE-lncRNAs according to the genome location classification in each comparison, separating up-regulation and down-regulation of those lncRNAs (Figure 2). A total of 60% of DE-lncRNAs with enhanced expression in WL-S after 7 days of hypoxia were classified to exonic overlap with reference on the opposite strand (x), whereas in WL-T accessions, only 18% (47) lncRNAs were assigned to that class (Figure 2). This may indicate a potential mechanism of gene regulation under the long-term waterlogging.

**Figure 3:**
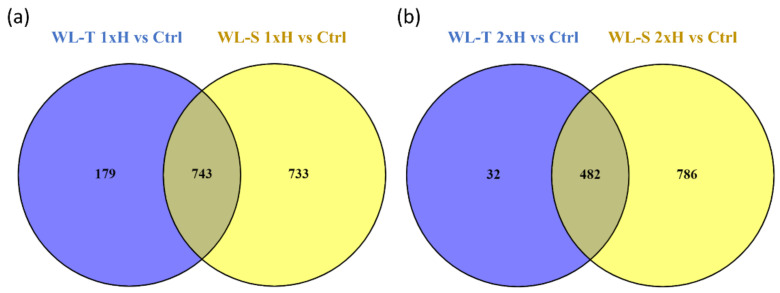
Venn diagrams showing the number of differentially expressed lncRNAs that are commonly and uniquely regulated in: (**a**) non-primed (1xH) and (**b**) primed (2xH) plants in two cucumber accessions, WL-T and WL-S.

**Figure 4:**
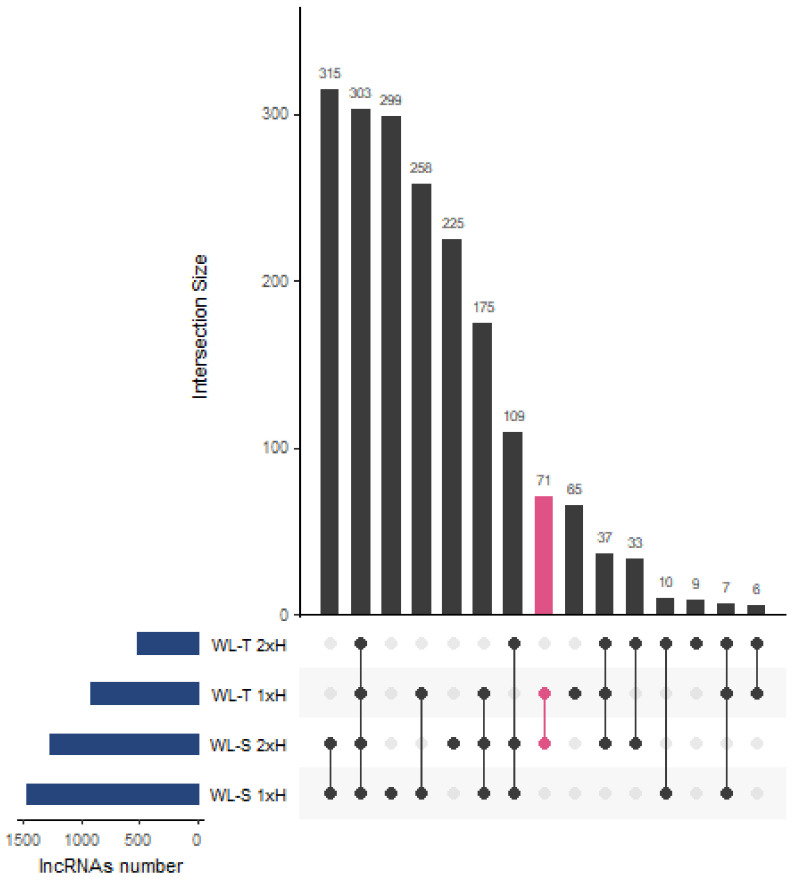
Upset plot presenting number of common and specific DE-lncRNA in four treatment groups in comparison to control conditions, i.e., non-primed (1xH) and primed (2xH) plants in two cucumber accessions, WL-T and WL-S. In pink, number of lncRNAs potentially involved in acquisition of long-term tolerance to waterlogging was shown.

In total, 922 and 1476 lncRNAs were differentially regulated in WL-T and WL-S, respectively, with 743 molecules shared in both accessions after 7 days of long-term waterlogging (non-primed, 1xH) (Figure 3a), and 482 after second exposure to stress (primed, 2xH) (Figure 3b). A total of 303 differentially expressed lncRNAs (DE-lncRNA) were identified across all treatment groups compared to control samples (Figure 4). The highest number of specific DE-lncRNAs was found in WL-S after a single hypoxia treatment (1xH), 299, whereas only 9 unique molecules were assigned to WL-T after second hypoxia treatment (2xH). We also indicated 71 DE-lncRNAs potentially involved in acquiring tolerance to oxygen deprivation, since they were regulated in non-primed in WL-T accession and in primed plants of WL-S (Supplementary Materials Data S6). Among those lncRNAs, the expression level of seven was enhanced, whereas inhibition was displayed by 61 of them in both accessions. TCONS_00009645 and TCONS_00019419 had different expression pattern between accessions, i.e., in WL-T they were up-regulated, while in WL-S they were down-regulated.

### 2.2. Target Genes for DE-LncRNAs

The genes located in the nearest location (down- and upstream) to the identified lncRNAs were considered as potential target genes. It was found that 3036 lncRNAs could potentially regulate expression of 2209 proximal genes (Supplementary Materials Data S1). In non-primed plants (1xH), 797 and 407 genes were identified as potentially regulated by DE-lncRNA in WL-T and WL-S accessions, respectively. In the case of primed plants (2xH), 402 and 1100 of genes were indicated as lncRNAs targets for WL-T and WL-S. 

**Figure 5:**
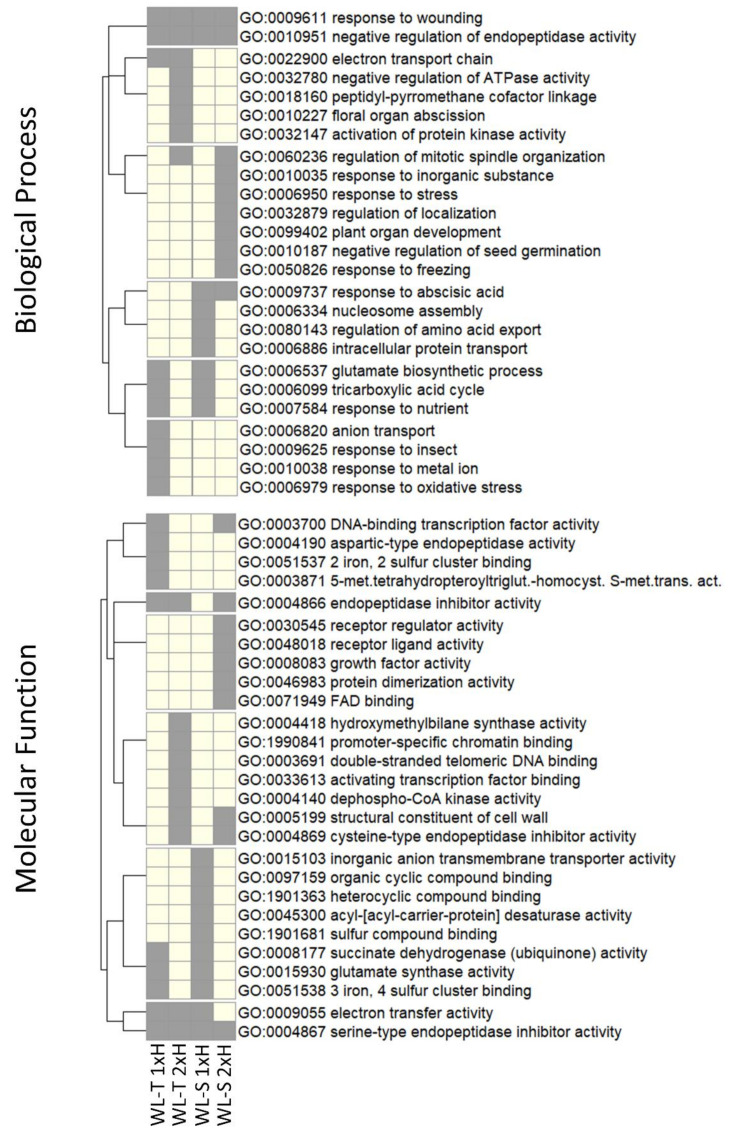
The top enriched GO categories in the Biological Process (BP) and Molecular Function (MF) determined for DE-lncRNAs target genes in non-primed (1xH) and primed (2xH) plants in two cucumber accessions, i.e., WL-T and WL-S under long-term waterlogging. Grey colour means presence of the term, whereas white colour indicates lack of statistically significant enrichment for the term.

GO enrichment analysis was conducted to determine the biological process and molecular functions (MF) in which potential targets genes are involved (Figure 5). The common significant GO terms for Biological Process (BP) in both accessions were response to wounding (GO:0009611) and negative regulation of endopeptidase activity (GO:0010951), whereas serine-type endopeptidase inhibitor activity (GO:0004867) was found to commonly enrich the Molecular Function (MF) category in response to oxygen deprivation in both accessions. DNA-binding transcription factor activity (GO:0003700) was an enriched term in non-primed WL-T and primed WL-S plants, highlighting its link to hypoxia stress in cucumber.

### 2.3. Validation of lncRNAs with Quantitative Real-Time PCR (QRT-PCR)

From the identified lncRNA molecules, eight were selected and validated with qRT-PCR (Table 1). The selected molecules revealed in the RNAseq data [7], among others, the opposite regulation in both cucumber accessions in non-primed plants. Additionally, they demonstrated different expression levels between non-primed (1xH) and primed (2xH) in both accessions, which may indicate that these molecules potentially play a role in waterlogging stress tolerance. 

**Figure 6:**
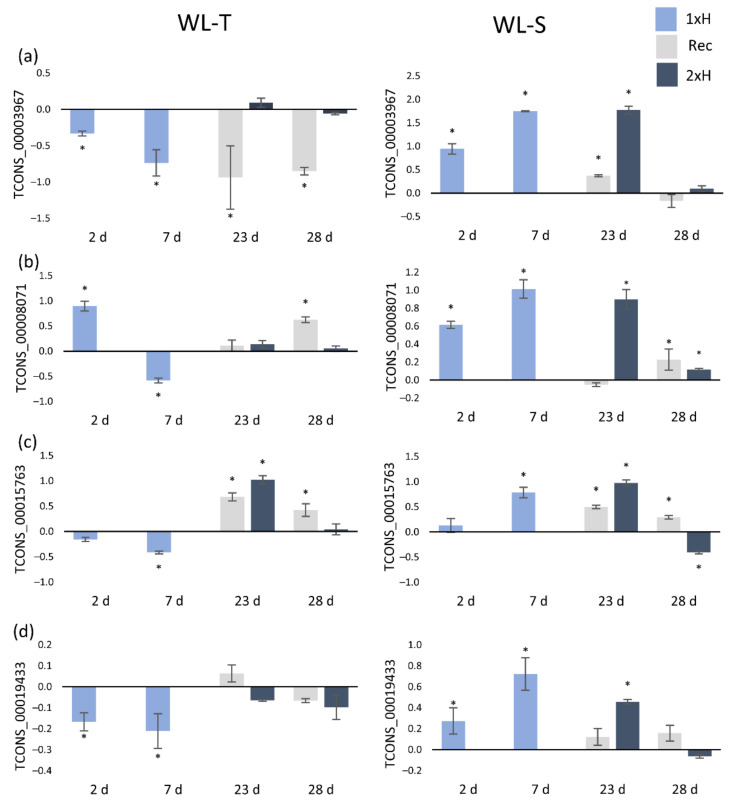
Expression profiles of four lncRNAs, i.e., TCONS_00003968 (**a**), TCONS_00008071 (**b**), TCONS_00015763 (**c**), and TCONS_00019433 (**d**), depicting differences in the expression levels in non-primed plants in WL-T and WL-S cucumber accessions under waterlogging stress. 1xH—non-primed plants, Rec—non-primed plants after 14-day recovery period, 2xH—primed plants. Data are expressed as the mean ± SD (standard deviation) of three independent biological replicates and three technical replications. Asterisks indicate a significant difference vs. control plants.

**Figure 7:**
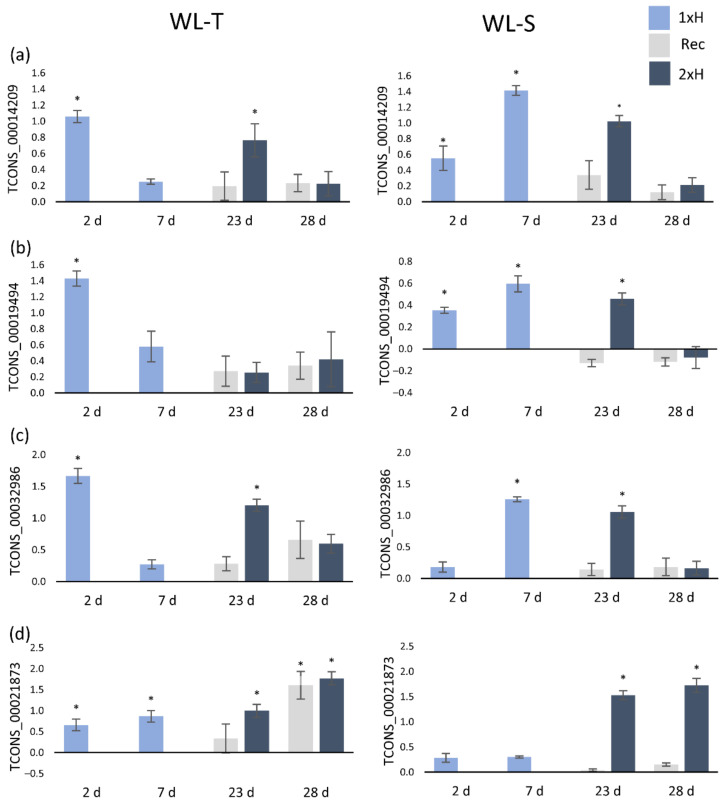
Expression profiles of four lncRNAs, i.e., TCONS_00014209 (**a**), TCONS_00019494 (**b**), TCONS_00032986 (**c**), and TCONS_00021873 (**d**) in WL-T and WL-S cucumber accessions under waterlogging stress. 1xH—non-primed plants, Rec—non-primed plants after 14-day recovery period, 2xH—primed plants. Data are expressed as the mean ± SD (standard deviation) of three independent biological replicates and three technical replications. Asterisks indicate a significant difference vs. control plants determined with Student *t*-test, *p* < 0.05.

Expression of all selected lncRNAs differentiates the studied cucumber accessions (Figure 6 and Figure 7). For example, after first waterlogging treatment (1xH) in WL-S, TCONS_00003967, TCONS_00008071, TCONS_00015763 and TCONS_00019433 were upregulated, while in the WL-T accession, expression levels of these lncRNAs were reduced in comparison to control conditions (Figure 6). In the cases of TCONS_00014209, TCONS_00019494 and TCONS_00032986, their expression was enhanced in non-primed plants of WL-S (after 7 days of first hypoxia treatment), whereas in the WL-T accession, expression remained unchanged in comparison to control plants (Figure 7a–c). Only one lncRNA (TCONS_00021873) was overexpressed after 7 days of stress in WL-T, whereas in WL-S, no regulation was detected (Figure 7d). 

Exposure of plants to another waterlogging treatment (2xH) led to up-regulation of these lncRNAs after 2 days in WL-S, while in WL-T, this could be observed for only some of them (TCONS_00015763, TCONS_00014209, TCONS_00032986 and TCONS_00021873) (Figure 6c and Figure 7a,c,d). In both primed WL-T and WL-S plants, i.e., after the second treatment, only the expression level of TCONS_00021873 was enhanced (Figure 7d). In WL-S, upregulation after 2 days of stress, and repression after 7 days were detected for all lncRNAs except TCONS_00021873 (Figure 6 and Figure 7a–c).

Validation by qRT-PCR indicated a specific overexpression of TCONS_00032986 and TCONS_00021873 after 2 days of stress induction only in WL-T (Figure 7c,d), which was not observed in WL-S accession in early response to stress. TCONS_00021873 was consistently expressed in WL-T at each time-point, whereas in WL-S, upregulation was detected only in primed plants (2xH) (Figure 7d).

### 2.4. miRNAs Involved in Response to Long-Term Waterlogging Stress

**Figure 8:**
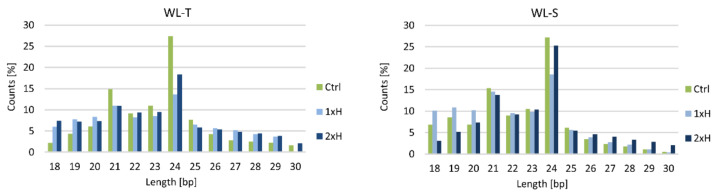
The length distribution for small RNA sequences WL-T and WL-S cucumber accessions of Ctrl, 1xH and 2xH experimental groups.

In total, 489,977,941 raw reads were obtained from 18 libraries, of which 252,796,468 reads were from WL-T accession and 237,181,473 reads were from WL-S accession libraries (Supplementary Table S1). As expected, the highest fraction of reads was 21–24 nucleotides long (Figure 8) in both accessions. Most reads were obtained from a library derived from WL-T control plants, and the length of these sequences was 24 bp (Figure 8). In WL-S, the number of reads of each length was similar between the libraries representing control (Ctrl) and primed plants (2xH). This relation was not observed for the number of reads in the DH2 accession. In total 684 miRNAs were detected in all samples (data available on request from the authors). 

### 2.5. Long-Term Waterlogging-Responsive Novel miRNAs in Cucumber

Identification of differently expressed miRNAs (DE-miRNAs) under oxygen deprivation was performed with edgeR programme. This led to the discovery of only 19 miRNAs presenting differential expression between non-primed (1xH), primed (2xH) and control (Ctrl) plants of both cucumber accessions, respectively. For 5 of 19 DE-miRNAs analysis with the use of ShortStack software confirmed these miRNAs to be novel (Supplementary Materials Data S7). 

**Figure 9:**
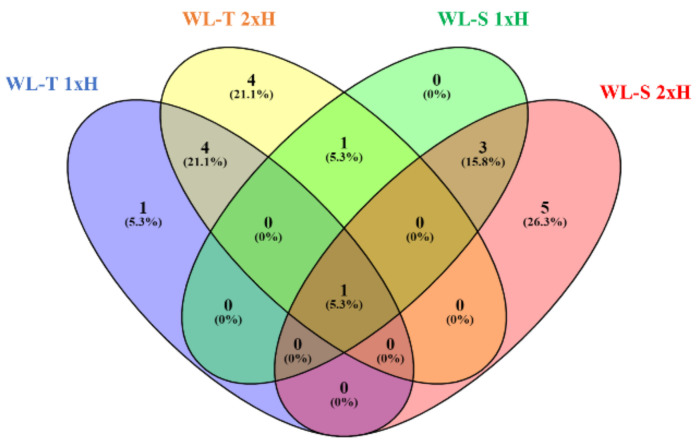
Venn diagram with common and specific miRNAs differentially expressed under hypoxic conditions (1xH, 2xH) in two cucumber accessions, i.e., WL-T and WL-S, compared to control.

**Figure 10:**
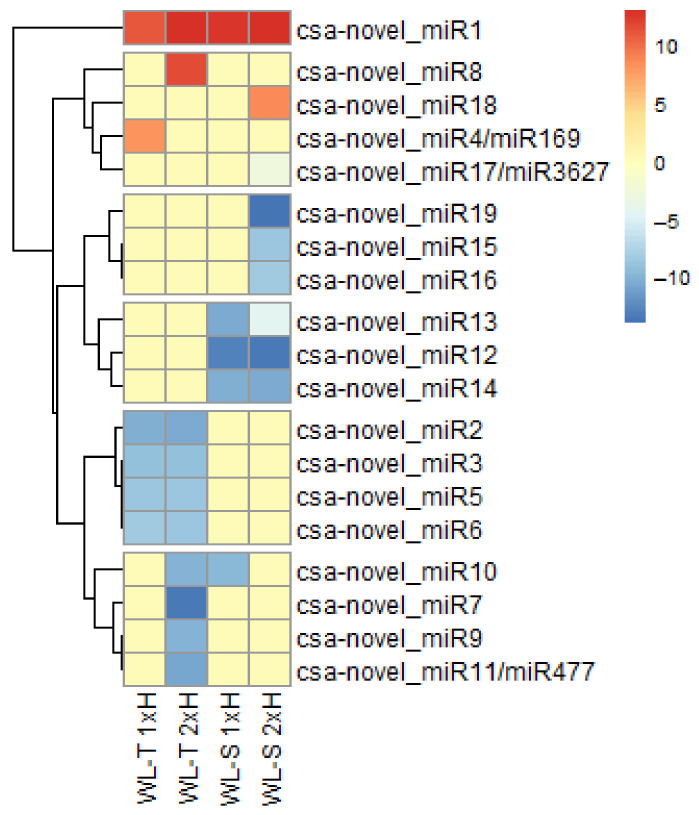
miRNAs differentially expressed in non-primed (1xH) and primed (2xH) cucumber WL-T and WL-S accessions under long-term waterlogging. The heatmap represents log_2_FC values in comparison to control conditions with FDR < 0.05.

The highest number of DE-miRNAs, 10, was found in primed plants of WL-T, whereas only five DE-miRNAs were depicted in non-primed plants of the WL-S accession (Figure 9). Of the 19 long-term waterlogging-responsive miRNAs, csa-novel_miR1 was strongly up-regulated in non-primed and primed plants of both accessions in response to long-term waterlogging (Figure 10). Three of the identified miRNAs, i.e., csa-novel-miR4/miR169, csa-novel_miR8 and csa-novel_miR18 molecules, were determined as being uniquely up-regulated in non-primed and primed plants of WL-T and primed plants of WL-S, respectively. Differences in expression levels of miRNAs between the treatments and accessions were observed, which may indicate a different response/tolerance to oxygen restricted stress.

The highest number of specific miRNAs was identified in primed plants (2xH) of the WL-S accession, while in non-primed plants, no specific miRNAs with differential expression were identified (Figure 9). For WL-T, one and four uniquely regulated miRNAs were found under long-term waterlogging in non-primed and primed plants, respectively.

The most up-regulated specific miRNA was csa-novel_miR8 (log_2_FC = 11.44) and regulation was observed in WL-T accession in primed plants (2xH). In turn, in primed plants of the WL-S accession, csa-novel_miR19 was mostly inhibited under oxygen deprivation (Figure 10).

### 2.6. QRT-PCR of miRNAs Involved in Long-Term Waterlogging

For qPCR assay, the miRNA with the highest expression level in response to waterlogging stress in both cucumber accessions was chosen (csa-novel_miR1). Additionally, we randomly selected miRNAs with stable expression between non-primed, primed and control plants, but with a high number of normalized reads in order to examine the early stage of response (csa-novel_miR20, csa-novel_miR21). To confirm hypoxic conditions, we chose csa-miR-394a [55]. 

**Figure 11:**
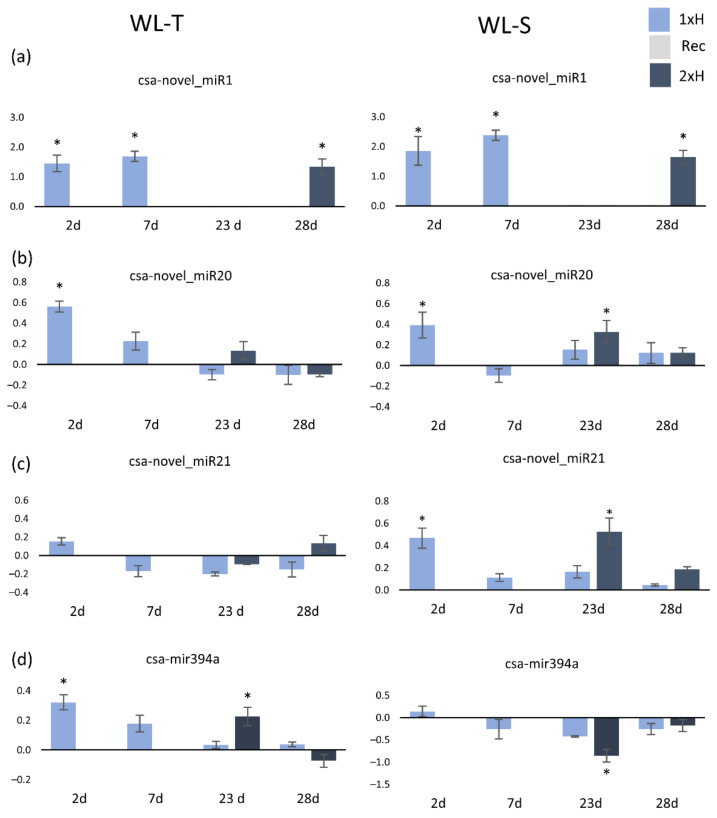
Expression level of three novel miRNAs, i.e., csa-novel_miR1 (**a**), csa-novel_miR20 (**b**), csa-novel_miR21 (**c**) and one known csa-miR394a (**d**) determined in non-primed and primed plants of WL-T and WL-S cucumber accessions. 1xH—non-primed plants, Rec—non-primed plants after 14-day recovery period, 2xH—primed plants. Data are the mean of three independent replicates. Asterisks indicate a significant difference vs. control plants determined with Student t-test, *p* < 0.05.

The enhanced expression of csa-novel_miR1 was detected in both accessions after 2 and 7 days in non-primed plants, while second treatment caused up-regulation only after 7 days of waterlogging (2xH) (Figure 11a). The expression of csa-novel_miR20 was enhanced after 2 days of first waterlogging treatment in both accessions; however, during the second treatment, expression of that miRNA was enhanced only in WL-S at the 2 day time-point (Figure 11b). Waterlogging treatment had an influence on differential expression of csa-novel_miR21 only in WL-S, with its up-regulation being observed after 2 days of first (1xH) and second stress (2xH) induction. In WL-T, differences between control and non-primed and primed, as well, were not detected (Figure 11c). Analysis of the expression pattern of csa-mir394a revealed increased expression levels on the second day of both waterlogging treatments in WL-T, whereas in WL-S, expression was inhibited after two days of the second treatment (Figure 11d). Long-term waterlogging did not have any impact on differential expression of csa-miR394a in cucumber. 

### 2.7. Interaction between LncRNAs and miRNAs

**Figure 12:**
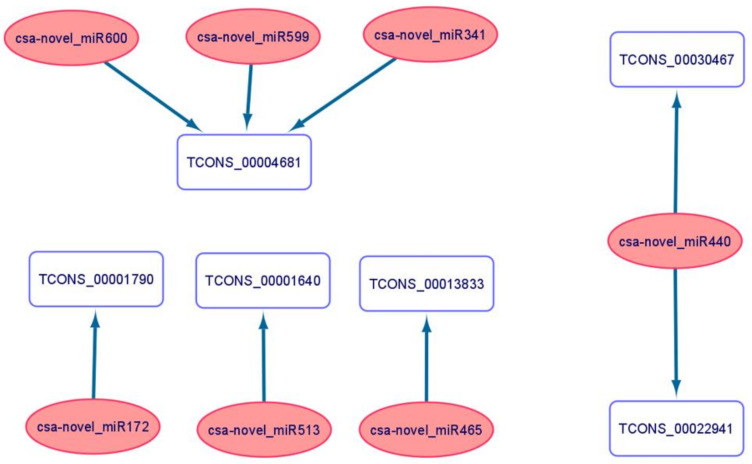
Interaction network analysis representing miRNAs (pink circles) with target lncRNAs (blue round rectangle).

A total of 208 DE-lncRNAs were revealed as potential targets for 207 miRNAs in cucumber under long-term waterlogging (Supplementary Materials Data S8). From the pool of all interactions, we selected the 71 lncRNAs that were possibly involved in acquiring tolerance to long waterlogging in cucumber to examine the potential role of miRNAs in their expression regulation. Six of the 71 lncRNAs were possibly targeted by seven miRNAs (Supplementary Table S2). TCONS_00004681 may be a target of three miRNAs. Five target lncRNAs were down-regulated in non-primed plants of WL-T and primed plants of WL-S, except TCONS_00030467, potentially depicted as one of two targets for csa-novel_miR440, as its expression was enhanced (Figure 12). 

**Figure 13:**
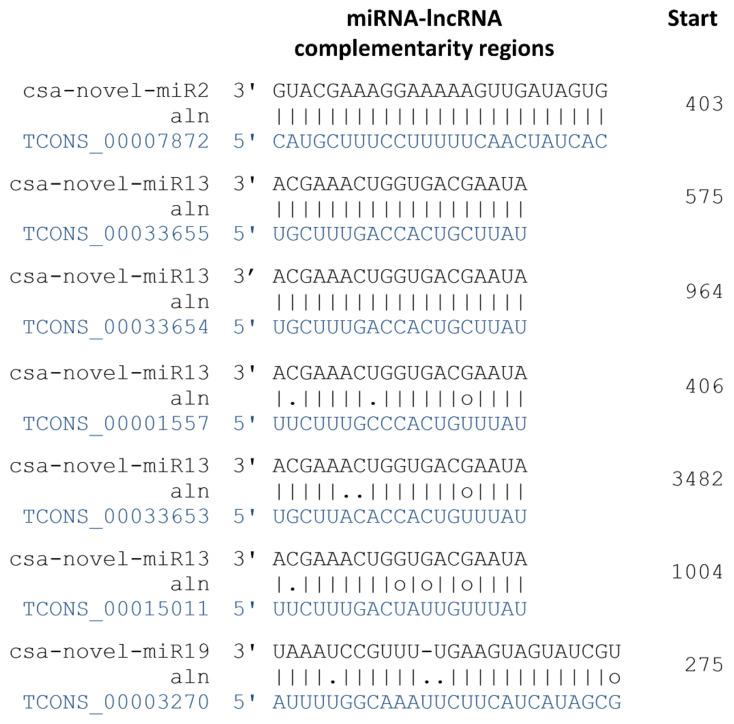
Identified endogenous target mimics (eTMs) of miRNAs. The ‘.’, ‘−‘, ‘o’, and ‘|’ represent mismatches, gaps, G:U pairs, and complementary bases, respectively.

Moreover, we found that five lncRNAs could act as endogenous target mimics (eTMs) for three DE-miRNAs. Interestingly, those miRNAs were down-regulated under long-term waterlogging (Figure 13). 

## 3. Discussion

Genes participating in the response to stresses can be regulated at transcriptional and post-transcriptional levels, among others, by ncRNAs. High-throughput sequencing assays make it possible, with better specificity, to detect those ncRNAs [32]. Understanding of the mechanisms participating in gene regulation under long-term waterlogging in cucumber will provide essential knowledge, making it possible to investigate accessions with enhanced tolerance to oxygen deprivation. 

### 3.1. LncRNAs Differentially Expressed under Long-Term Waterlogging 

About 37% of all identified lncRNAs in cucumber under long-term waterlogging treatment were classified in terms of their location in the genome into a group of molecules that exonically overlap with a target gene on the opposite strand, which may indicate their role in the silencing of gene expression. The highest number of overexpressed lncRNAs classified to this class was identified in WL-S in non-primed plants. 

Differences in the number of regulated lncRNAs between tolerant and sensitive genotypes were also determined in *Brassica napus* L. under drought stress and re-watering [56]. A higher number of DE-lncRNAs was indicated in the sensitive genotype, similar to results obtained in our research. These results confirm differences between cucumber accessions in response to long-term waterlogging. The number of DE-lncRNAs in primed plants of WL-S accession decreased in comparison to the non-primed plants, meaning fewer genes was regulated. This may suggest that another exposure to stress is less harmful for the cucumber plants.

LncRNAs can regulate expression of genes located nearby [57]. In our study, 2209 genes were predicted as being potentially targeted by 3036 lncRNAs. GO enrichment analysis was performed for target genes of DE-lncRNAs in non-primed and primed cucumber accessions under long-term waterlogging. We observed a diversity of GO terms enriched by target genes in each comparison, confirming differences in response to hypoxia in both cucumber accessions. In non-primed plants of WL-T accession, target genes were specifically enriched in processes involved in response to oxidative stress (GO:0006979) caused by reactive oxygen species (ROS) production. Overrepresentation of ROS can lead to damage of cellular components, such as membrane lipids, and as a consequence to cell death, so it is important to activate the mechanism to prevent this [58]. Balance between ROS production and its scavenging is related to waterlogging-tolerant species [59]. Regulation of genes involved in the response to oxidative stress under oxygen deprivation was also observed in tolerant cucumber accession [60]. 

DE-lncRNAs target genes in non-primed plants of the WL-S accession were specifically enriched with respect to nucleosome assembly (GO:0006334), regulation of amino acid export (GO:0080143), and intracellular protein transport (GO: 0006886). Exposure of the plants to unfavourable environmental conditions must immediately be responded to by changes at the biochemical and physiological levels caused by the regulation of gene expression, whereby a fundamental role is played by nucleosome assembly. Highly conserved histone chaperones are involved in nucleosome assembly, leading to remodelling of the chromatin structure during transcription or DNA replication [61,62]. Enrichment in the nucleosome assembly category was noted only in the non-primed plants of WL-S, which may indicate that chromatin organization is more advanced upon oxygen deprivation in WL-S than in WL-T. Functional prediction showed that target genes potentially regulated by lncRNAs in non-primed plants of WL-S were also specifically enriched in processes connected with transport inside the cells and between the plant organs in order to maintain homeostasis in the cells and to transport, among other things, a reduced form of nitrogen [63,64]. 

qRT-PCR validation of selected lncRNAs was consistent with results obtained using the RNA-Seq approach. We depicted four lncRNAs, i.e., TCONS_00003967, TCONS_00019433, TCONS_00032986, and TCONS_00021873, that can be used for cucumber differentiation regarding their tolerance to hypoxia stress on just the second day of long-term waterlogging. Differences in lncRNA expression levels determined by qRT-PCR between accessions have also been established in Chinese cabbage under heat stress conditions [65]. In our study, TCONS_00003967 and TCONS_00019433 were up-regulated in WL-S, whereas TCONS_00032986 and TCONS_00021873 presented over-expression in WL-T. In the context of priming and acquiring tolerance to hypoxia, we observed that TCONS_00021873 was up-regulated in WL-T, whereas in WL-S it was not differentially expressed, but its expression was enhanced in primed plants, meaning that priming resulted in regulation of that lncRNA molecule. TCONS_00021873 was classified as an intergenic lncRNA, so its function needs to be further elucidated, for example by genome editing. 

Six among eight validated lncRNAs were classified according to the location in the genome as exonic overlapped with a reference on the opposite strand, which potentially means that expression of co-located target-genes may be regulated by those lncRNAs. We showed that TCONS_00003967 could potentially regulate the xyloglucan endotransglucosylase/hydrolase gene, TCONS_00008071 can target the malate dehydrogenase gene, TCONS_00015763—the syntaxin gene, TCONS_00019433 can influence the gene-encoded 26S proteasome non-ATPase regulatory subunit, TCONS_00019494 can be located close to the ATP-dependent RNA helicase gene, and finally, TCONS_00032986 can regulate the transcription initiation factor TFIID subunit 1-A gene. 

Our results revealed a down-regulation of lncRNA (TCONS_00003967), potentially targeting the xyloglucan endotransglucosylase/hydrolase gene, in non-primed WL-T and up-regulation of this lncRNA in WL-S, and additionally, we found a down-regulation of xyloglucan endotransglucosylase/hydrolase in the WL-S accession in the RNA-Seq assay, suggesting that TCONS_00003967 inhibits expression of the target genes due to its antisense location. Overexpression of gene-encoded xyloglucan endotransglucosylase/hydrolase results in enhanced flooding tolerance in soybean [66] by modifying the cell wall architecture, leading to adaptation to waterlogging [67]. 

lncRNA, i.e., TCONS_00008071, which potentially regulates the malate dehydrogenase gene was down-regulated in the WL-T and up-regulated in the WL-T accession in non-primed plants. Compared to RNA-Seq data, the malate dehydrogenase gene presented diverse expression patterns, which may indicate that the lncRNA TCONS_00008071 regulates target gene expression at the transcriptional level by inhibition. In research described by Qi et al. [47] on the effects of hypoxia on gene expression level in tolerant accessions of cucumber, malate dehydrogenase was down-regulated; however, data in that research were established at 24 h after start of waterlogging, and it can be said that the expression level of malate dehydrogenase changes with the duration of oxygen deprivation, and is enhanced with long-term waterlogging treatment, which was also detected in barley after 3 weeks of stress [68]. Malate dehydrogenase is also involved in tolerance to abiotic stresses, such as cold and salt by, among others, reducing the levels of superoxide anion (0^2−^) [69]. 

In our studies, we also determined the syntaxin gene to be a potential target, up-regulation of which led to tolerance to oxidative stress [70]. We observed up-regulation of TCONS_00015763 in non-primed plants of the WL-S accession and down-regulation of this lncRNA in non-primed plants of the WL-T and primed plants of the WL-S accession, resulting in the expression level of the target gene in this group, i.e., syntaxin, being detected as overexpression in non-primed WL-T plants and primed WL-S plants. This suggests that primed WL-S plants may acquire tolerance to oxidative stress, causing, among other things, damage to carbohydrates, amino-acids and lipid membranes during hypoxia stress in plants [58]. In its interaction with lncRNA-target genes, TCONS_00015763 may regulate the expression of target genes through inhibition of expression. Further study is required regarding TCONS_00015763 as a syntaxin regulator in acquiring tolerance to hypoxia stress. 

The same expression pattern in terms of lncRNA–target gene interaction as that found in the previously described genes was also observed for TCONS_00019433, which can potentially regulate the 26S proteasome non-ATPase regulatory subunit (*RPN1A*) gene. RPN1A is needed for optimal growth and response to stress (https://www.uniprot.org/uniprot/Q9SIV2, accessed on 28 May 2021). This lncRNA–target gene interaction may be another example of the influence of priming on enhancing tolerance to long-term waterlogging. In the literature, there is lack of strict evidence of the participation of the 26S proteasome non-ATPase regulatory subunit in the response to waterlogging in cucumber, so this is the first information found regarding the potential role of TCONS_00019433–26S proteasome non-ATPase regulatory subunit in developing tolerance to hypoxia in cucumber. 

It was found that TCONS_00019494 can influence the expression of the ATP-dependent RNA helicase gene. Overexpression of that lncRNA was only observed in non-primed plants of the WL-S accession, and simultaneously, expression of target-genes was also enhanced. This suggests that TCONS_00019494 does not affect the target gene through inhibition of expression. This mechanism needs to be further elucidated. 

Deeper analysis and exploration are needed to understand interactions of lncRNAs and potential target genes, especially those located close to protein-coding genes, in terms of their expression and influence on tolerance to hypoxia stress in cucumber. Understanding of the influence of miRNA on lncRNA and mRNA regulation is really challenging, since one miRNA can regulate more than one target gene, which consequently affects several pathways in plants [71].

### 3.2. miRNAs Involved in Response to Long-Term Waterlogging

Although there is more and more research on the involvement of miRNAs in response to abiotic stresses, when it comes to cucumber, the miRNAs were discovered in the context of oxygen deprivation only in the early-stage response to stress [28]. Hence, our research is the first report providing information about the involvement of miRNA in the response to long-term waterlogging in cucumber. Moreover, we contributed information on the regulation of miRNAs in primed plants. 

In this work, 18 libraries of cucumber roots were constructed for non-primed, primed and control plants of the WL-T and WL-S cucumber accessions. The most abundant miRNAs peak size was 24 bp for all experimental groups, which is consistent with results obtained in tolerant cucumber accessions in hypocotyls under waterlogging stress [28]. The same size of miRNA was also detected in cucumber in response to powdery mildew [72]. This may suggest that 24 bp is a typical size for cucumber miRNAs [55].

Bioinformatic analysis revealed only 19 miRNAs with differential expression level in response to long-term waterlogging. All of them were considered to be novel. The obtained miRNAs were analysed in terms of interaction with lncRNAs. 

We validated three novel miRNAs and one based on the literature by qRT-PCR assay. The obtained results were in agreement with data provided by small RNA-Seq. Expression of csa-novel_miR1 confirmed the sequencing data, i.e., csa-novelmiR1 was overexpressed in non-primed and primed plants of both accessions. There were no differences in the expression between cucumber accessions, which may suggest that this miRNA is involved in response oxygen deprivation in cucumber regardless of tolerance level. Analysis of target genes prediction depicted that csa-novel_miR1mir1 can regulate the auxin response factor, which is involved in morphological changes of roots [73]. Auxins are one of the most common phytohormones participating in response to hypoxia stress in plants, and the auxin response factor regulates genes involved in auxin signalling pathways through binding to their promoters [74,75].

Csa-novel_miR21 was only expressed in the WL-T accession in the early stage of waterlogging treatment. Target gene prediction revealed that csa-novelmiR21 may regulate gene encoded scarecrow-like protein 15, which participates in root growth under waterlogging, for example in *Brassica napus* [76]. 

Csa-novel_miR20 was involved in the response to oxygen deprivation in the early stage of both non-primed accessions, its overexpression was also detected after 2 days of the second waterlogging treatment of WL-S. Target gene analysis predicted homeobox-leucine zipper protein ATHB-15 as a potential target of csa-novel_miR20. *ATHB-15* with interaction with miRNA is involved in cell modification, and in consequence, being inhibited by miRNA, causes elongation of roots. It is known that *ATHB-15* is a mir166 target [77], so our results may provide a new specific sequence of mir166 in cucumber. 

qRT-PCR validation of miR394 determined up-regulation of this miRNA in early response to the first and second waterlogging treatments of the WL-T accession, which was consistent with the results obtained by Xu [28]. In WL-S, the expression of mir394 was stable in non-primed plants, during the second treatment, after 2 days, expression was inhibited. These results may suggest that mir394 is expressed only in tolerant cucumber accessions, and is involved only in early response to waterlogging. 

miRNAs can affect target genes in a positive or negative way, and this may lead to tolerance to abiotic stresses [78]. 

### 3.3. Interaction between LncRNAs and miRNAs

In the current research, lncRNAs involved in response to oxygen deprivation were identified, but we also wanted to determine the molecules potentially involved in acquiring tolerance to that stress. As a result, we indicated 71 molecules regulated in non-primed of WL-T and primed plants of WL-S, assuming them to have potential roles in waterlogging tolerance. Among the 71 lncRNAs, TCONS_00019419 was classified as being completely matched to the intron of UDP-glycosyltransferase 1 (Csa4M051380) and TCONS_00020999 exonically overlapped transcription initiation factor TFIID subunit on its opposite strand (Csa4M652030), suggesting that those targets can be regulated by the two identified lncRNAs. Two lncRNAs classified as intergenic, i.e., TCONS_00010998 and TCONS_00003889, were simultaneously up-regulated in non-primed plants of WL-T and primed plants of WL-S, and they could potentially be involved in the gene regulation network, hence their specific role in the response to hypoxia in cucumber needs to be elucidated. Seven out of 71 lncRNAs can potentially be targeted by miRNAs, resulting in instability of lncRNA. This mechanism has been reported in drought stress in *Solanum lycopersicum* [79], as a response to giberrelin in *Populus tamentosa* [80], and in the flower development of *Cicer arietinum* [81]. 

LncRNA can act as endogenous target mimics of miRNA, resulting in blocking of the interaction between miRNA and its target gene by, for example, increasing expression [82], and this interaction is considered to be the most crucial in the regulatory gene networks of plants [83]. This mechanism has been reported under heat stress in Chinese cabbage [65], phosphate (Pi) deficiency in *Arabidopsis* [84], and also in species closely related to *Cucumis sativus*, e.g., *Cucumis melo*, in response to powdery mildew [85]. We found that five of the lncRNAs bound the miRNA binding sites of the csa-novel_miRNAs. Additionally, all of those miRNAs were down-regulated under waterlogging stress, meaning that those lncRNAs acted as miRNA decoys, inhibiting their expression.

These interactions showed that the regulatory pathways of those genes are complex under long-term waterlogging stress in cucumber, and an understanding of all of the processes is needed in order to evaluate accessions tolerant to oxygen deprivation. However, information regarding lncRNAs in response to long-term waterlogging in cucumber, to the best of our knowledge, is still not available, so this research provides essential knowledge about the participation of non-coding RNA upon oxygen deprivation in the root zone in cucumber. Additionally, this research made it possible to indicate lncRNAs involved in acquiring tolerance to hypoxia stress via the application of long-term waterlogging priming. The identification of lncRNAs in this research filled a gap in knowledge regarding the involvement of lncRNAs in long-term waterlogging response. Additionally, lncRNA–miRNA interactions were established, which is a big step forward in understanding complex interactions with respect to gene regulation under long-term waterlogging stress in cucumber.

## 4. Materials and Methods

### 4.1. Plant Growth and Stress Induction

Seeds of cucumber double haploid lines, DH2, waterlogging tolerant (WL-T), and DH4, waterlogging sensitive (WL-S), with confirmed response to hypoxia stress [7,54], were obtained from a Polish breeding company KHiNO Polan (Krakow, Poland). Plant cultivation and experiment conditions were the same those described in Kęska et al. [7]. Samples for all assays were collected from the same experiment to eliminate the influence of different conditions in order to be able to compare results from each assay with one another. Plants were divided into 3 groups: (1) plants untreated with waterlogging (Ctrl), (2) plants whose root zones were waterlogged for 7 days (1xH, non-primed), and (3) plants waterlogged for a second time after a 14-day recovery period (2xH, primed).

### 4.2. Sample Collection and RNA Extraction

**Figure 14:**
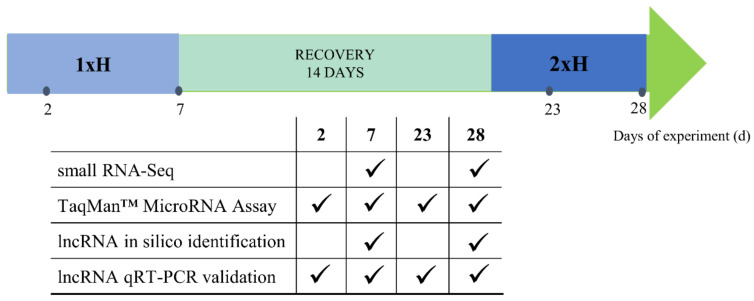
A scheme describing the experiment duration and time-points (days) of sample collection for small RNA-Seq assay, TaqMan^TM^ MicroRNA Assay, lncRNA in silico identification and lncRNA validation by qRT-PCR.

For small RNA-Seq assay, roots of both cucumber DH lines, WL-T and WL-S, were collected after 7 days of the first hypoxia stress treatment (1xH 7 d) and after 7 days of the second stress induction (2xH 28 d) (Figure 14). For TaqMan^TM^ MicroRNA Assay and lncRNA validation by qRT-PCR, samples were additionally collected after 2 days of both hypoxia stress treatments, in order to detect the expression levels of selected miRNAs and lncRNAs in early phase of stress response. Twelve root samples from plants of control group (Ctrl) were collected at 7 d and 28 d of the experiment (6 from each time-point) and separately divided into 3 independent biological replicates, where one biological replicate was pooled of roots from 4 plants: 2 plants from 7 d and 2 from 28 d. For plants of 1xH and 2xH treatment groups, 3 independent biological replicates were prepared, each pooled with roots from 4 plants. Before freezing in liquid nitrogen, roots were carefully washed in water in order to remove peat substrate. Total RNA extraction from all samples was performed with Direct-zol RNA MiniPrep Plus (Zymo Research, Irvine, CA, USA) following the manufacturer’s instructions. To remove DNA contamination, obtained RNA extracts were treated with 1 U µL^−1^ RNase-free *Dnase* I (Thermo Fisher Scientific, Waltham, MA, USA) and 40 U µL^−1^ of RiboLock RNase Inhibitor (Thermo Fisher Scientific, Waltham, MA, USA). The gel electrophoresis under denaturing conditions was performed to assess RNA quality and quantity. The A260/A280 ratio and RNA integrity number (RIN) were determined by a Bioanalyzer 2100 (Agilent 2100 Bioanalyzer; Agilent Technologies, Palo Alto, Santa Clara, CA, USA) and samples with RIN > 7 were chosen for further analysis.

### 4.3. Small RNA Library Preparation, Sequencing and Bioinformatic Analysis of Small RNA Sequencing Data

For small RNA-Seq sequencing, in total, approximately 1 μg total RNA was initially used for BGISEQ-500 library construction. Eighteen libraries (2 cucumber accessions, (i.e., WL-T DH2 and WL-S DH4) × 3 treatments (Ctrl, 1xH, 2xH) × 3 experimental replicates) retaining information about the transcript direction (strand specific) were prepared. Then, these libraries were subjected to next-generation SE50 (single-end 50 bp) NGS sequencing for small RNAs using the BGISEQ-500 platform (BGI, Shenzen, China). The sequencing data for WL-T DH2 (Ctrl, 1xH, 2xH) and WL-S DH4 (Ctrl, 1xH, 2xH) were deposited in the NCBI Sequence Read Archive (SRA, http://www.ncbi.nlm.nih.gov/Traces/sra/) under BioProject No. PRJNA721283. 

Analysis of small RNA-Seq data was conducted with CAP-miRSeq [86]. The first stage of the analysis was to check the quality of the reads with FastQC ver. 0.10.1 (http://www.bioinformatics.babraham.ac.uk/projects/fastqc). Then the reads were mapped with Bowtie program ver. 0.12.7 (http://bowtie-bio.sourceforge.net/index.shtml) to the cucumber reference genome—ASM407v2 (https://www.ncbi.nlm.nih.gov/assembly/GCF_000004075.2/). The number of reads mapped to individual genes was counted using the HTseq program ver. 0.6.1 (https://htseq.readthedocs.io/en/release_0.10.0/).

The miRDeep2 program [87,88] detected known and novel miRNAs (not present in the database) using data available in the miRBase database ver. 22.1 (http://www.mirbase.org/), reference genome and sequence structural properties. Additionally, the ShortStack program [89] with default settings was applied to confirm predicted novel miRNAs in cucumber. Identified miRNAs were mapped to the cucumber reference genome—ASM407v2 (https://www.ncbi.nlm.nih.gov/assembly/GCF_000004075.2/).

Identification of significantly differently expressed miRNAs (DE-miRNAs) was performed using the edgeR package ver. 3.20.1 [90]. The following comparisons for both cucumbers (WL-S and WL-T) were conducted: 1xH vs. Ctrl, 2xH vs. Ctrl. The miRNAs with FDR ≤ 0.05 were considered as differentially expressed between comparisons. 

Applying the psRNATarget program (http://plantgrn.noble.org/psRNATarget) [91], potential target sites were detected for differentially expressed miRNAs, matching their sequence with cDNA of the reference genome ASM407v2). The following parameters were implemented, i.e., E ≤ 3, maximum energy of unpairing (UPE) the target site was set as 25 kcal, penalty for G:U pair was 0.5 and flanking length around the target site was chosen as 17 nucleotides upstream and 13 nucleotides downstream.

### 4.4. LncRNA Identification

For the prediction of lncRNAs involved in response to hypoxia stress in cucumber, the assembled transcriptome from PRJNA678740 project deposited in the NCBI Sequence Read Archive (SRA, http://www.ncbi.nlm.nih.gov/Traces/sra/) for Ctrl, 1xH and 2xH of WL-T and WL-S accessions [7] was compared against a reference cucumber transcriptome (ftp://cucurbitgenomics.org/pub/cucurbit/genome/cucumber/Chinese_long/v2) using Cuffcompare ver. 2.2.1 from Cufflinks package implementing the -R and -C options. Then, transcripts were processed according to the following steps: (a) discarding transcripts < 200 nucleotides in length to eliminate miRNA, rRNA, tRNA, snoRNA, siRNA, (b) filtering transcripts containing open reading frames (ORFs) encoding proteins greater than 100 amino acids using TransDecoder [92], (c) elimination of transcripts classified as coding by CPC (coding potential calculator) [93,94], (d) filtering out the sequences described in the Rfam database [95] identified using the BLASTN program (ver. 2.2.26) (E < 1 × 10^−5^).

Identified lncRNAs were classified according to their genomic context, using Cuffcompare methodology, i.e., ‘=’—complete match of intron chain, ‘j’—potentially novel isoform (fragment), at least one splice junction is shared with a reference transcript, ‘i’—a transfrag falling entirely within a reference intron, ‘o’—generic exonic overlap with a reference transcript, ‘u’—unknown, intergenic transcript, ‘x’—exonic overlap with reference on the opposite strand. 

GO enrichment analysis of the targeted genes of the mRNA:miRNA and mRNA:lncRNA interactions was implemented using the topGO R package ver. 2.38.1 [96].

### 4.5. Identification of the LncRNA–miRNA Interactions

Identified lncRNAs were checked as to whether they could potentially be targeted by miRNAs or play a role as endogenous target mimicry for miRNAs. An online server psRNATarget was used to predict target sites for miRNAs on lncRNAs with the same parameters set as for miRNAs target gene correlation. TAPIR (http://bioinformatics.psb.ugent.be/webtools/tapir/) [97] was used for prediction of endogenous target mimics (eTMs) with mfe ratio ≥ 0.6 [98]. Networks for the miRNA:lncRNA interactions were built using Cytoscape ver. 3.8.2 (https://cytoscape.org/).

### 4.6. Quantitative Real-Time PCR (QRT-PCR) Validation of LncRNAs

lncRNA selection for validation by qRT-PCR was based on the results of differential expression analysis of transcripts obtained with the edgeR package ver. 3.20.1 [90]. We randomly selected lncRNAs that, firstly, differentiated accessions from each other after 7 days of waterlogging (1xH) and, secondly, allowed to differ non-primed and primed plants (Table 1). Specific pairs of primers (forward and reverse) for amplification of the selected lncRNAs were designed using Primer3 ver. 3-0.4.0 (http://bioinfo.ut.ee/primer3-0.4.0/) and IDT PrimerQuest (https://eu.idtdna.com). Obtained oligonucleotides were additionally checked with the IDT PrimerQuest program for their physicochemical properties, in order to eliminate the likelihood of the formation of secondary structures (hairpins) as well as heterodimers between the primers of the same pair. List of primers used in the assay can be found in Supplementary Material Table S3. Quantitative real time PCR assay was performed as described in our previous work [7]. Three independent biological replicates, each with three technical replicates, were run for every sample/lncRNA combination. The expression levels of the selected lncRNAs were determined using the ∆∆Ct comparative method with the use of two selected reference genes (*Act*, *Tua*) (Supplementary Table S3) and standard curves [99]. The data were then logarithmized (log_2_FC) in order to confront the experimentally obtained expression level of the selected lncRNAs with the results of the RNA-Seq analysis.

### 4.7. Quantitative Real-Time PCR (QRT-PCR) of miRNAs

The expression profiling of selected miRNAs was performed using real-time qPCR. An amount of 2 ng of each RNA sample was processed, strictly following the protocol described in TaqMan^TM^ Small RNA Assay (Publication Number 4364031) using TaqMan^TM^ MicroRNA Reverse Transcription Kit (No 4366596, Applied Biosystems^TM^, Foster City, CA, USA) for cDNA synthesis with miRNA-specific stem-looped RT primers provided in the kit. qRT-PCR reactions were conducted in 96-well plates with 0.67 μL of RT product with TaqMan PCR master mix (No 4304437) and TaqMan probes for each miRNA in a total volume of 10 μL. A QuantStudio™ 3 Real-Time PCR System (Applied Biosystems, Foster City, CA, USA) was used to perform the qRT-PCR reactions with U6 as an endogenous control. Three biological replicates were considered, with 3 technical replicates for each of them. The relative expression level of each miRNA was analysed by the ∆∆Ct comparative method.

### 4.8. Statistical Analysis

Comparison between treatments at each time-point and the control condition from the same timepoint was performed. Student’s *t*-test with *p* < 0.05 was used to compare changes in relative expression levels of lncRNAs and miRNAs to control conditions as reference. Data are presented as the mean value of three biological replicates ± SD for each treatment and time points. The analysis was performed using Statictica ver. 12 (Statsoft).

## Figures and Tables

**Table 1:** List of lncRNA molecules selected for validation using qRT-PCR on the basis of the RNAseq data [7].

lncRNA	WL-T		WL-S		Class Code	Nearest Gene	Gene Description	Gene Ontology
	1xH *	2xH **	1xH	2xH				
TCONS_00003967	−4.57	ns ***	6.93	ns	x	Csa1M422990.1	Xyloglucan endotransglucosylase/hydrolase	-
TCONS_00008071	−4.66	ns	7.44	ns	x	Csa2M174150.1	Malate dehydrogenase	BP: GO:0006099
TCONS_00015763	−8.06	ns	6.62	−4.41	x	Csa3M782680.1	Syntaxin, putative	BP: GO:0009737
TCONS_00019433	−7.42	ns	7.13	ns	x	Csa4M054300.1	26S proteasome non-ATPase regulatory subunit	-
TCONS_00014209	ns	ns	5.51	ns	u	-	-	-
TCONS_00019494	ns	ns	8.87	ns	x	Csa4M063450.1	ATP-dependent RNA helicase, putative	MF: GO:0097159, GO:1901363
TCONS_00032986	ns	ns	6.07	ns	x	Csa7M357030.1	Transcription initiation factor TFIID subunit 1-A	-
TCONS_00021873	5.23	7.34	ns	3.50	u	-	-	-

* Differences in expression between non-primed (1xH) and unstressed plants (Ctrl), ** differences in expression between primed (2xH) and unstressed plants (Ctrl), *** ns—no statistically significant differences in expression.

## Data Availability

Data are contained within the article or Supplementary Material. The RNA-Seq datasets generated for this study are deposited in the NCBI under BioProject PRJNA678740 and can be found in the GenBank Short Read Archive (SRA) under Acc. No. SSR13083584 to SSR13083607. The small RNA-Seq datasets are deposited in the NCBI under BioProject PRJNA721283.

## Supplementary Materials

The following are available online at https://www.mdpi.com/article/10.3390/ijms22158197/s1, Supplementary Materials Data S1: Identified lncRNAs, Supplementary Materials Data S2: Differentially Expressed lncRNAs in non-primed 1xH WL-T vs. Ctrl, Supplementary Materials Data S3: Differentially Expressed lncRNAs in primed 2xH WL-T vs. Ctrl, Supplementary Materials Data S4: Differentially Expressed lncRNAs in non-primed 1xH WL-S vs. Ctrl, Supplementary Materials Data S5: Differentially Expressed lncRNAs in primed 2xH WL-S vs. Ctrl, Supplementary Materials Data S6: lncRNAs potentially involved in acquiring tolerance to hypoxia in cucumber, Supplementary Materials Data S7: Differentially Expressed miRNAs in all comparisons, Supplementary Materials Data S8: lncRNAs potentially targeted by miRNAs, Supplementary Table S1: Summary of sequencing results for 18 libraries of miRNA molecules identified for the WL-T and WL-S cucumber accessions, Supplementary Table S2: Complementary pairing of lncRNAs targeted by miRNAs in cucumber under long-term waterlogging, Supplementary Table S3: Primers used in qRT-PCR assay.
