# Supplementary material for: Waterlogging-Stress-Responsive LncRNAs, Their Regulatory Relationships with miRNAs and Target Genes in Cucumber (Cucumis sativus L.)
Source: Int J Mol Sci. 2021 Jul 30;22(15):8197. doi: 10.3390/ijms22158197 (PMC8348067; doi:10.3390/ijms22158197)
Supplement: Supplementary file 1 [file ijms-22-08197-s001.zip › ijms-1285913-Supplementary.pdf]

Supplementary Table S1. Summary of sequencing results for 18 libraries of miRNA molecules identified for the DH2 and DH4 accessions.

Accession	Treatment	Biological replicate	Reads with a length in range 18-45 bp	Reads mapped to the genome of *C. sativus* - **ASM407v2**
DH2 WL-T	Ctrl	1	29 120 653	23 761 223 (82%)
		2	28 397 136	22 524 447 (79%)
		3	29 148 988	24 009 000 (82%)
	**Total**		**86 666 777**	**70 294 670 (81%)**
	1xH non-primed	1	28 586 089	23 534 135 (82%)
		2	29 419 466	22 496 743 (76%)
		3	26 756 942	23 009 521 (86%)
	**Total**		**84 762 497**	**69 040 399 (81%)**
	2xH primed	1	29 287 792	22 395 141 (76%)
		2	25 020 112	19 933 103 (80%)
		3	27 059 290	21 937 204 (81%)
	**Total**		**81 367 194**	**64 265 448 (79%)**
DH4 WL-S	Ctrl	1	25 774 212	19 836 599 (77%)
		2	26 493 178	20 797 107 (78%)
		3	28 072 883	22 680 168 (81%)
	**Total**		**80 340 273**	**63 313 874 (79%)**
	1xH non-primed	1	22 871 866	18 836 746 (82%)
		2	20 473 332	16 966 500 (83%)
		3	28 349 182	23 630 961 (83%)
	**Total**		**71 694 380**	**59 434 207 (83%)**
	2xH primed	1	28 709 963	22 878 578 (80%)
		2	28 529 322	22 086 921 (77%)
		3	27 907 535	22 023 716 (79%)
	**Total**		**85 146 820**	**66 989 215 (79%)**

Supplementary Table S2. Complementary pairing of lncRNAs targeted by miRNAs in cucumber under long-term waterlogging.

miRNA	Target	E*	Alignment
csa-novel_miR172	TCONS_00001790	2.5	miRNA 21 CCACUGUCUCUCUCUCUUUCG 1<br>::: ::::::::::::::.<br>Target 1105 UCUGA-AGAGAGAGAGAAAGU 1124
csa-novel_miR341	TCONS_00004681	2.5	miRNA 24 UAACGAUUUUAUCAGGAAGUAAAG 1<br>: ::.:::.::: ::::::::<br>Target 383 GAGGGUAGAAUGGUCAUUCAUUUC 406
csa-novel_miR513	TCONS_00001640	3.0	miRNA 24 GUGCUAGAAGUUGGUUUAUUAUGA 1<br>:: .:::.::::::::::<br>Target 1598 UUUACUCCAUAACUAAAUAAUACU 1621
csa-novel_miR600	TCONS_00004681	3.0	miRNA 24 UCGAAUGUACGUGGAGUUGAUUAA 1<br>::.:::.:: :::.:::::::::<br>Target 960 AGUUUAUAUACACUUCAACUAAUC 983
csa-novel_miR599	TCONS_00004681	3.0	miRNA 24 UCGAAUGUACGUGGAGUUGAUUAA 1<br>::.:::.:: :::.:::::::::<br>Target 960 AGUUUAUAUACACUUCAACUAAUC 983
csa-novel_miR465	TCONS_00013833	3.0	miRNA 24 UGCUUUUGUACCAGCUAUCAUUUA 1<br>:.: :::.:: ::: :::::::::<br>Target 679 AUGCAAAUAUAGUCAAUAGUAAAU 702
csa-novel_miR440	TCONS_00030467	3.0	miRNA 25 UGAGAGAGAGAAAAGAAAGAGAGAC 1<br>::.:.:.:::::::.:.:::<br>Target 585 AACCUUUUUUUUUUCUUUUUUUCUU 609
csa-novel_miR440	TCONS_00022941	3.0	miRNA 25 UGAGAGAGAGAAAAGAAAGAGAGAC 1<br>:::.:.::::.:::.:.:.::<br>Target 1357 GUAAUCUUUUUUUUUUUUUUUUUUG 138

Supplementary Table S3. Primers used in qRT-PCR assay.

**lncRNA**		**Primers sequences F: 5' - 3' R: 5' - 3'**	**Product length (nt)**	**Primers efficacy (%)**
TCONS_00003967	F	TTGAGGATCTGAGCACGACC	171	104.16
	R	TCCGAACTTCCATCTCCATCT		
TCONS_00008071	F	GGTGCCCTGGAAGGATATTAAG	101	101.26
	R	GAACTCCTGAGGTTGGTGATTT		
TCONS_00015763	F	TGTGAAGGGTCTTGCTCTGTT	146	104.32
	R	GAACGATTTATTCTCCTCTTCCCCC		
TCONS_00019433	F	TGGGTTCTCCAAAGCAGAAGG	134	99.25
	R	AGAGATTTCTTCCTCCATCCCC		
TCONS_00014209	F	TGGAAGAATCTATTGGAGCAATGAA	141	104.5
	R	AACCAACCGGTAATCCTTCACC		
TCONS_00019494	F	TCAGAGACAACAGGGAAGCC	145	100.02
	R	TCGTGGTAATGGTGTGGGTT		
TCONS_00032986	F	ACGACCAGATGGATCACCAA	118	101.79
	R	CAACCTTCTTGTCTGACCCC		
TCONS_00021873	F	TGGTGAATGAGGGAGAAGA	100	100.7
	R	ACCCTTGCAGCTTCATTT		
**Reference genes**				
Act (*actin*) (Qi et al. 2012)	F	TGGACTCTGGTGATGGTGTTA	150	101.5
	R	CAATGAGGGATGGTGGAAAA		
Tua (*tubulin alpha chain*) (Wang et al. 2014)	F	ACGCTGTTGGTGGTGGTAC	210	104.4
	R	GAGAGGGGTAAACAGTGAATC		
